# Treatment seeking behavior and associated factors of suspected dengue fever among Shan people in eastern Shan special region IV, Myanmar: a cross-sectional study

**DOI:** 10.1186/s12913-020-05163-z

**Published:** 2020-04-16

**Authors:** Hui Liu, Jian-Wei Xu, Zadan Ai, Yan Yu, Bian Yu

**Affiliations:** 1 Yunnan Institute of Parasitic Diseases, Yunnan Provincial Key Laboratory of Vector-borne Diseases Control and Research, Yunnan Provincial Collaborative Innovation Center for Public Health and Disease Prevention and Control, Pu’er City, 665000 China; 2 Institute of Pathogens and Vectors, Dali University, Xiaguang, 671000 China; 3 The Hospital of Eastern Shan Special Region IV, Mengla Township, Myanmar

**Keywords:** Dengue fever, Treatment-seeking behaviors, Influencing factors, Shan people, Myanmar

## Abstract

**Background:**

Dengue fever (DF) is a rapidly spreading mosquito-borne disease along the China-Myanmar border. Understanding treatment-seeking behaviors (TSBs) and associated factors of suspected DF patients in local communities helps to improve health services via promoting prompt treatment, improving patients’ prognosis, finding DF information and timely response to DF foci.

**Methods:**

A combination of qualitative semi-structured in-depth interview (SDIs) included 18 key-informants, and quantitative household questionnaire survey (HHSs) involved 259 households was carried out to investigate TSBs and associated factors of suspected DF patients in the Eastern Shan Special Region IV (ESSR4), Myanmar.

**Results:**

The key informants mentioned that most of their fellow villagers did not seek treatment in public health facilities first. The HHS questionnaires were distributed to household heads, and 241 of the 259 HHS respondents were valid after data auditing. Only 102 (43.2%) household heads reported that their family sought treatment for suspected DF at a public health facility immediately; 111 (46.1%) respondents said that they chose self-medication first. The adjusted odds ratio of multivariate logistic analysis (MLA) predicting household heads’ first seeking healthcare at a public hospital were 1.91 (95%CI: 1.03–3.53) for those who knew DF and 5.11 (95%CI: 2.08–12.58) for those who regarded DF as a deadly disease, indicating that families who knew DF and regarded DF as a deadly disease were more likely to seek treatment for suspected DF at a public health facility immediately.

**Conclusion:**

The inappropriateness of treatment-seeking behaviors for suspected DF hinders the improvement of the patient prognosis and dengue control in ESSR4, Myanmar. People’s awareness of the potential seriousness of DF is a factor influencing appropriate healthcare-seeking behavior among Shan People.

## Background

In comparison with 50 years ago, the worldwide incidence of dengue has risen 30-fold [1, 2]. Dengue is now ranking as one of the most critical global mosquito-borne viral diseases and is endemic in over 100 countries [2, 3]. For many countries, dengue is becoming a threat to their public health, and further adversely impacting their health services and economies [4]. The South-East Asia Region (SEAR) is a focus of dengue fever. Over 70% of the worldwide population at risk of dengue lives in the South-East Asia Region and Western Pacific Region of World Health Organization [5]. In Indonesia, dengue peaks around every 6 to 8 years. Improved treatment for dengue fever (DF) has decreased the case fatality rate by approximately half with each decade since 1980 [6]. Dengue has replaced malaria to become another threat to public health along the China-Myanmar border [7, 8] as malaria has been successfully controlled [9]. More prompt and proper interventions are needed now because of the unavailability of anti-dengue drugs and low efficacy of current dengue vaccines [10, 11]. Early diagnosis and effective supporting treatment for DF can reduce transmission and improve patient prognosis. Some studies document that early supportive treatment can reduce the fatality rate from 20 to 1% or less [5, 12, 13]. Treatment-seeking behaviors (TSBs) are critical for those who have a suspected dengue infection. Patients must have the intention and the means to seek medical care early in the disease attack. Therefore, more studies are needed to investigate local health beliefs and practices, TSBs, and access to care concerning dengue fever to identify challenges and opportunities in diagnostics and treatment [13]. Early diagnosis and effective supporting treatment for DF requires appropriate infrastructure and resources, and also active engagement of communities [6]. Data on treatment-seeking behaviours and affecting factors for suspected DF patients are rare in the Greater Mekong Subregion (GMS). To address this gap, by collaborating with local institutes, we conducted a cross-sectional study to investigate treatment-seeking behaviours and associated factors among the Shan People in the Eastern Shan Special Region IV (ESSR4), Myanmar.

## Methods

### Study design

This cross-sectional study adopts a mixed-methods approach to collect data, combining qualitative semi-structured in-depth interviews (SDIs) and quantitative household questionnaire surveys (HHSs). In this study, the definition of treatment- seeking behaviours is what the families would expect to do and whether they would want to seek treatment if any household member had a fever that was suspected possibly to be DF. Based on DF incidence in 2017, two types of villages with and without DF cases were deliberately sampled in Mongla Township, ESSR4 of Myanmar. The study is a part of the project of the Shan people’s knowledge, attitude and practices among Shan People in the Eastern Shan Special Region IV (ESSR4), Myanmar [14]. To ensure that our sample was sufficient to address the main aims of the study, a small percentage was used to calculate the appropriate sample size. Based on standard value normal distribution at 95% confidence levels, an estimated 20% of adult people who know that mosquitoes transmit dengue virus and 5% precision, a sample size of 250 heads of household for the questionnaire survey was obtained [15]. The form of questionnaire survey included family wealth index (Table 1), symptoms of suspected DF and treatment seeking behaviours for suspected DF.

**Table 1:** Components for the construction of the family wealth index (FWI)

Family wealth index	Housing characteristics	Transportation tools	Family belongings
1 Poorest	Bamboo walls and sheet iron roofs	None	None or chickens
2 Mid low	Wood walls and sheet iron roofs	Bicycles	Pigs or goats
3 Middle	Brick walls, wood girders and terracotta roofs	Motorcycles	Cattle or horses
4 Mid high	Brick concrete walls and terracotta roofs	Tractors	TV sets or refrigerators
5 Least poor	Steel and concrete	Cars	Shops

### Study site and population

**Fig. 1:**
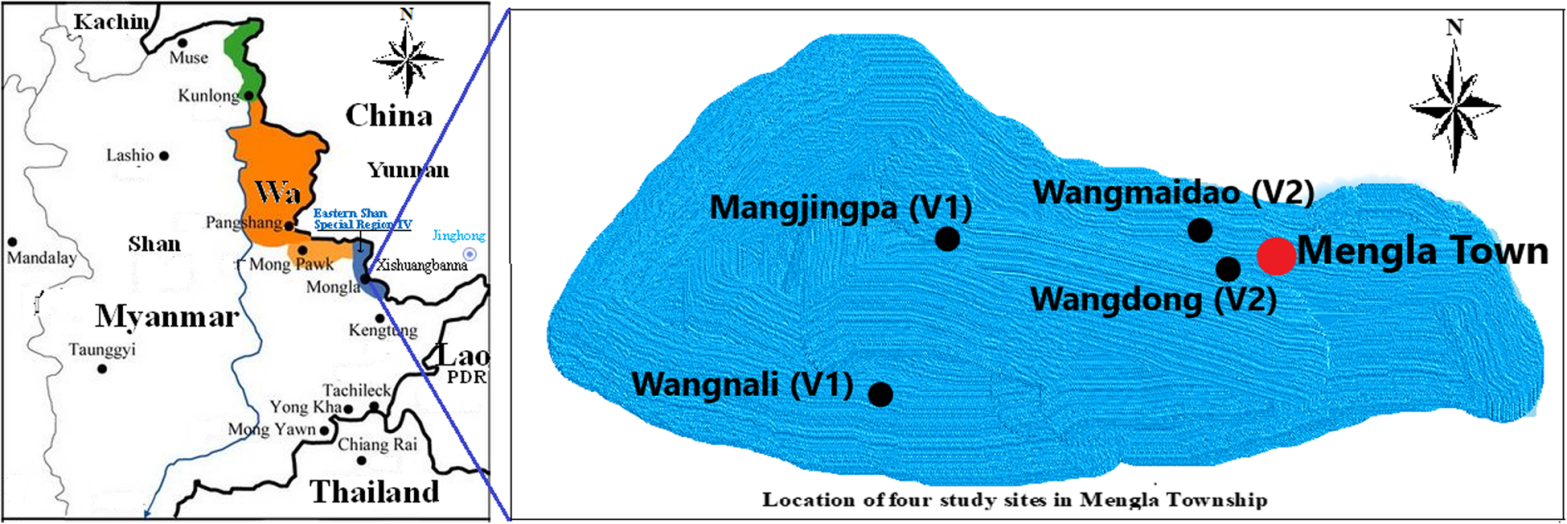
Location of study site and neighboring areas: The blue is the study site, the Eastern Shan Special Region IV, Myanmar. Neighbouring areas are Shan Special Region II (locally named Wa), Myanmar; Yunnan Province, China; Lao PDR; Thailand. The figure was generated by using the drawing tool of Microsoft Windows 10 software

The ESSR4 is about 80 km from Kengtung, the capital of Eastern Shan State of Myanmar and borders with Xishuangbanna Prefecture, China (Fig. 1) [16]. The council of ESSR4 administraters Mongla Township, Nanban and Selei County where there is a population of about 110,000, most of whom are Shan people. The Shan (known as Dai in China, Thai Yai in Thailand and Lao in Lao PDR) is one of the mainstream ethnicities in the GMS. The hospital of ESSR4 is the sole health facility that can do laboratory-based diagnosis and treatment for DF. After obtaining the permission of the Bureau of Health of ESSR4, the hospital disclosed to us that it reported a total of 114 DF cases in 2017.

Two Villages without DF cases (V1) and two villages with DF cases (V2) were selected for the study, respectively. The criteria for selecting V1 were: (1) there were no laboratory confirmed DF cases in 2017; (2) all households were Shan people; (3) there were at least 300 households together. The criteria for selecting V2 were that one village was that with the highest DF incidence and another one was a middle DF incidence in village in the ESSR4, as well as the criterion (2) and (3) for V1. Based on these criteria and suggestion of the Bureau of Health of ESSR4, the researchers and the hospital reached a consensus to select the four villages of Mangjingpa, Wangnali, Wangmaidao and Wangdong as the study locations (Fig. 1). There were no DF cases in 2017 in Mangjingpa and Wangnali (V1) with 867 residents and 147 households, and there were 45 DF cases in Wangmaidao and Wangdong (V2) with 876 residents and 150 households from January to November, 2017.

### Field survey

The language commonly used for communication is Chinese, which is one of the two official languages (Burmese and Chinese) of the Council of ESSR4, Myanmar. Thus, the SDI guidelines (Additional File 1) and HHS questionnaires (Additional File 2) were developed in two versions of Chinese and Shan language. Investigators from the Hospital of ESSR4 who understand both the Shan language and Chinese conducted the field survey. The investigators discussed the questions with respondents in Ethnic Shan language and then filled the questionnaire in Chinese. The SDIs were administered to 18 key informants including village leaders, community health worker and representatives, who were supposedly more knowledgeable about dengue. The investigators discussed with the key informants about treatment-seeking behaviors that most of their fellow villagers usually carried out when they experienced fever that was suspected possibly to be DF and also related influencing factors. In the HHSs, household heads were selected as respondents. The household list of each selected village was obtained through the four Villager Committee offices, and then households were sampled by simple computer randomization. The investigators visited house by house to tell the head of each sampled household about the purpose of the project, the topic, and the type of questions to be asked. After an oral informed consent was obtained, a questionnaire was administered to them to collect quantitative data on treatment-seeking behavior and associated factors [17–19]. Family wealth index (FWI) in the questionnaire was determined by household characteristics [17, 18], such as housing, walls and roofs, and assets, such as bicycles, and then classified into five groups, ranked from 1 to 5, representing the poorest to the Least poor (Table 1).

### Data management and analysis

Data of both SDIs and HHSs were entered in Microsoft Excel 2007. One researcher coded records of the qualitative SDI based on the contents of the questions and then entered the information into cells in Microsoft Office Excel 2007. The same content records were combined with code sequencing. The records of each content were analysed by two independent researchers to generate themes first, and then the two researchers’ findings were discussed and compared to finalize the findings [14]. Data of HHSs were analysed in Epi Info 7.2. The percentage and their 95% confidence interval (CI) were calculated for their first treatment actions. A chi-squared test was used to compare the percentages of each aspect of behavior between villages with DF and without DF cases. A multivariate logistic analysis (MLA) was used to assess the association of expected treatment-seeking at public hospitals first and potential influencing factors. In the MLA model, the outcome variable coded with “1″ is that a household expected to seek treatment at public hospitals (STPHs) first if a family member experienced fever that was suspected possibly to be DF. The independent variables were characteristics of household heads and their families, including perceptions, beliefs, and knowledge of DF [16–18]. In the case that a respondent’s skipping a question led to missing data, the contents of the question were excluded from analyses.

## Results

### Characteristics of households

The 18 key informants of SDIs comprised nine males and nine females ranging from 32 to 54 years old. The HHS questionnaires were administered to a total of 259 household heads, and 241 questionnaires were considered valid after auditing. The age median of these respondents was 48.3 (range: 18–54) years. Females accounted for 143 (59.3%) heads of households. Only 27 (11.2%) HHS respondents had formal school education ranging from 1 to 10 years. Most of the families (195) involved in the study belonged to the category of ‘less poor’ (i.e. with FWI 4 or 5, see Table 3).

### Treatment-seeking behaviours

The SDI results showed that most of local people investigated did not seek treatment from health facilities during the first 1 or 2 days of fever. Most villagers chose to use Guasha (scraping) therapy and Shan traditional herbal medicine at first if they had a fever or headache. They would not visit local health facilities until suffering severe illness or symptoms that could not be relieved. Consistent with the interviewing results, HHS results showed that 46.1% (111/241) of households chose to use self-medication at first; only 42.3% (102/241) sought treatment in public hospitals. Significantly, 6.2% (15/241) said they prioritized using traditional Shan medication and 8.7% (21/241) reported using other treatment resources. Behaviors of self-medication were various, ranging from Guasha therapy to Shan traditional herbal medicine, the use of over-the-counter drugs, and family stored drugs. Moreover, 5.8% (14/241) of households said that they did not take any action first, and they just waited to see if they could autonomously recover within 2 or 3 days (Table 2).

**Table 2:** The first treatment-seeking behaviours of suspected dengue cases in Eastern Shan Special Region IV, Myanmar

Treatment-seeking behaviors	Total No. (%, 95CI), *n* = 241	No. (%, 95CI) in V1, *n* = 124	No. (%, 95CI) in V2, *n* = 117	*P*-value
Public hospitals	102 (42.3, 36.0–48.8)	61 (49.2, 40.1–58.3)	41 (35.0, 26.5–44.4)	0.037
Self medication	111 (46.1, 39.6–52.6)	51 (41.1, 32.4–50.3)	60 (51.3, 41.9–60.6)	0.147
Traditional Shan medication	15 (6.2, 3.5–10.1)	9 (7.3, 3.4–13.3)	6 (5.1, 1.9–10.8)	0.677
Others	21 (8.7, 5.5–13.0)	6 (4.8, 1.8–10.2)	15 (12.8, 7.4–20.3)	0.049
No action	14 (5.8, 3.2–9.6)	4 (3.2, 0.9–8.1)	10 (8.5, 4.2–15.2)	0.136

*Abbreviation*: *V1* village without DF cases, *V2* village with DF cases, *95%CI* 95% confidence interval

### Influencing factors of treatment-seeking behaviours

Household head’s awareness of DF was identified by MLA as an independent factor associated with first seeking treatment at a public hospital. Adjusted odds ratio (AOR) of household heads who know DF was 1.91 (95%CI: 1.03–3.53), and who regarded DF as a deadly disease was 5.11 (95%CI: 2.08–12.58) in comparison with those who did not know DF and who did not regard DF as a deadly disease, respectively. This difference shows that the families having higher DF awareness were more likely to seek treatment in public health hospitals when having a fever (Table 3). Previous DF experience of a community was possibly a marginal influencing factor. In villages with DF (V2), the proportion of families of STPHs was significantly lower (*P* = 0.037) than that in villages without DF (V1) (Table 2). The crude odds ratio was 0.56 (95%CI: 0.33–0.94), but after MLA controlling for potential confounding, the AOR was 0.59 (95%CI: 0.30–1.17) (Table 3).

**Table 3:** Characteristics of households and factors associated with first seeking treatment in a public hospital, Eastern Shan Special Region IV, Myanmar

Independent variables	No. respondents	No. STPHs (%)	Crude OR (95% CI)	***P*** values	Adjusted OR (95% CI*)	***P*** values
**Villages**						
**DF cases**	117	41 (35.0)	0.56 (0.33–0.94)	0.027	0.59 (0.30–1.17)	0.132
**Non DF**	124	61 (49.2)	1		1	
**Sex of the household heads**						
**Male**	98	44 (44.9)	1.18 (0.70–1.98)	0.532	1.17 (0.69–2.00)	0.558
**Female**	143	58 (40.6)	1		1	
**Age of the respondents (years)**						
**18–45**	95	42 (44.2)	1.14 (0.56–1.91)	0.632	1.31 (0.63–2.77)	0.473
**46–54**	146	60 (41.1)	1		1	
**School education**						
**Yes**	27	11 (40.7)	0.90 (0.40–2.04)	0.803	0.43 (0.13–1.43)	0.170
**No**	208	90 (43.3)	1		1	
**Family wealth index**						
**4–5**	195	84 (43.1)	1.18 (0.61–2.27)	0. 620	1. 53 (0.62–3.78)	0.360
**1–3**	46	18 (39.1)	1		1	
**Belief of the Buddha protecting good people**						
**Yes**	191	87(45.6)	1.84 (0.94–3.6)	0.076	1.87 (0.89–3.97)	0.104
**Non**	48	15 (31.2)	1		1	
**Belief of natural factors influencing human health**						
**Yes**	146	61 (41.8)	0.92 (0.54–1.57)	0.759	0.92 (0.53–1.59)	0.755
**No**	89	39 (43.8)	1		1	
**Belief of sound hygiene being helpful for people’s health**						
**Yes**	203	88 (43.4)	1.24 (0.59–2.60)	0.577	0.94 (0.42–2.09)	0.864
**No**	34	13 (38.2)	1		1	
**Heard about DF**						
**Yes**	83	46 (55.4)	2.35 (1.36–4.05)	0.002	1.91 (1.03–3.53)	0.040
**No**	153	53 (34.6)	1			
**Regard DF a deadly disease**						
**Yes**	63	38 (60.3)	4.40 (2.13–9.09)	< 0.001	5.11 (2.08–12.58)	< 0.001
**No**	74	19 (25.7)	1		1	
**Know fever as one of DF symptoms**						
**Yes**	46	25 (54.3)	1.85 (0.89–3.84)	0.098	1.96 (0.93–4.17)	0.078
**No**	125	48 (38.4)	1		1	
**Perceive DF transmissible**						
**Yes**	41	23 (56.1)	2.01 (1.01–4.00)	0.047	1.72 (0.80–3.67)	1.168
**No**	175	68 (38.9)	1		1	
**Family income source**						
**Others**	158	60 (38.0)	0.67 (0.34–1.31)	0.245	0.75 (0.36–1.54)	0.433
**Agriculture**	44	21 (47.7)	1		1	
**Family decision**						
**Wife or co-decision**	132	66 (50.0)	2.00 (1.12–3.41)	0.011	1.50 (0.81–2.79)	0.200
**Husband**	102	34 (33.3)	1		1	

For all variables, there were a total of 241 respondents who answered questions on seeking treatment, unless otherwise indicated;

*Abbreviation*: *DF* dengue fever, *STPHs* seeking treatment at public hospitals first, *OR* odds ratio, *95%CI* 95% confidence interval

## Discussion

Dengue is becoming a major threat to public health globally [20]. With the lack of effective antiviral therapies for DF [21], early diagnosis and timely treatment influence the prognosis of DF patients. In contrast, delay in proper treatment can lead to complications or to severe dengue [22]. The study demonstrates that treatment-seeking behaviors regarding suspected DF are inappropriate in the ESSR4, Myanmar. Most respondents did not initially visit public health facilities when having a fever (Table 2). Perceived awareness of DF significantly influenced their TSBs. Similar results were presented in Venezuela [22] and Malaysia [23]. The results indicate that it is critical to raise people’s awareness of appropriate treatment-seek practices. In Myanmar, five Special Regions are mostly administered by local ethnic minority authorities along the China-Myanmar border. As a result, health services provided by the Myanmar central government cannot fully cover these regions, and thereby health services there are somewhat limited [14, 17, 19]. Consequently, international investment and collaboration are urgently needed for dengue control there.

In the ESSR4, only the hospital of ESSR4 can perform laboratory-based diagnosis and treatment. DF is becoming a new threat to public health after the China Global Fund to fight AIDS, Tuberculosis and Malaria project has successfully reduced the malaria burden along the China-Myanmar border [9]. However, funding is still not enough to control DF. Information, education, and communication on DF have not been effectively performed in ESSR4. In this study, only 19.1% (46/241) of HHS respondents knew DF and listed fever as one of DF symptoms (Table 3). The results of this study show that local people cannot recognize DF symptoms and the value of seeking proper diagnosis and effective supporting treatment in formal health facilities. When effective antiviral therapies of DF are still unavailable, DF treatment can largely rely on symptom-relief-based supporting therapy. Some Shan traditional therapies such as Guasha (scraping) and herbal drugs are said to be able to relieve fever, headache and other symptoms. Traditional medical practices and home remedies were also widely perceived and experienced as efficacious for treating DF in Malaysia [22]. In this situation, without antiviral therapies of DF, research on efficacy and limitation of Shan traditional therapies for DF might be an interesting topic that needsto be explored. Such studies can provide evidence for efficacy, safety and limitation on the traditional medication. When results of researches can provide solid evidences on risks of traditional therapies, communication of updated evidence to the public would help improve seeking treatment for DF and dengue intervention.

This study has an obvious limitation. Due to the limited facility of laboratory-based diagnosis, the number of confirmed DF cases was limited. This study could only investigate treatment-seeking behaviors of suspected DF, namely, what the families would expect to do and where they would expect to seek treatment if a household member experienced fever that was suspected possibly to be DF. However, this kind of treatment-seeking intention study would be also helpful for further intervention in ESSR4 of Myanmar or other regions with a similar context.

## Conclusion

The results of this study indicated that treatment-seeking behavior of suspected DF patients is not appropriate in the ESSR4 of Myanmar. Local people’s awareness of DF is a major influencing factor in the situation of lacking sound knowledge about DF among Shan people. In the setting of a weak health system, international collaboration and support are urgently needed.

## Supplementary information

**Additional file 1.**

**Additional file 2.**

## Data Availability

The datasets used and/or analyzed during the current study are available and can be obtained from the corresponding author on reasonable request.

## Abbreviations

DF: Dengue fever
SDI: Semi-structured in-depth interview
HHS: Household questionnaire survey
CI: Confidence interval
TSB: Treatment-seeking behaviors
GMS: Greater Mekong Subregion
V1: Village Mangjingpa and Wangnali where there were not dengue fever cases in 2017
V2: Wangmaidao and Wangdong where there were dengue fever cases in 2017
FWI: Family wealth index
MLA: Multivariate logistic analysis
AOR: Adjusted odds ratio
ESSR4: Eastern Shan Special Region IV
