# Supplementary material for: Treatment seeking behavior and associated factors of suspected dengue fever among Shan people in eastern Shan special region IV, Myanmar: a cross-sectional study
Source: BMC Health Serv Res. 2020 Apr 16;20:318. doi: 10.1186/s12913-020-05163-z (PMC7164341; doi:10.1186/s12913-020-05163-z)
Supplement: Supplementary file 1 — Additional file 1. [file 12913_2020_5163_MOESM1_ESM.doc]

**The Guidelines of semi-structured in-depth interview on Dengue Fever**

**Introduction**

The purpose of our investigation today is to understand the knowledge of dengue among the local people, and to publicize the knowledge of dengue prevention and treatment. Dengue fever is a viral disease transmitted by mosquitoes. It is the key to prevent mosquito bites. Therefore, it is necessary to remove the water accumulated in containers such as pots and pans indoors and outdoors, because the mosquitoes that transmit dengue fever like to grow in the water accumulated in pots and pans; reducing the growth of mosquitoes (called Aedes mosquitoes) can prevent dengue fever. Understanding treatment-seeking behaviors and associated factors of suspected DF patients in local communities can help to improve health services via promoting prompt treatment, improving patients’ prognosis, finding DF information and response to DF foci timely. You can refer the fallowing question during interviewing.

1. Which diseases are most common in your village? Please list by commonly prevalence.
2. What do you think is the cause of illness? Why do you get sick? (from the perspective of social, economic and religious beliefs, investigate the views and beliefs of each ethnic groups on life and health, and interpret the impact of social and economic development on the prevention and control of infectious diseases and people's health from the perspective of social culture). For example:
3. Do you think people with poor economic conditions, such as poor food and nutrition, are more likely to get sick?
4. Do you think that you will get sick because of retribution if you don't honor your parents and old people and do bad things?
5. Do you have any religion? What is the religion if you had?
6. Do you think that if you do good deeds and accumulate virtue, you will be blessed by gods (Buddha, Virgin Mary, Jesus Christ or spiritual things) and your body will be better?
7. Do you think the weather, water and forest will affect people's health? If so, what is more likely to cause illness? Such as air, weather and forest.
8. Do you think living in a place with poor sanitation is more likely to get sick? If so, can sanitation prevent disease? What sanitary conditions do you think are less likely to get sick?
9. Let the key informants list symptoms by asking: Have you heard of dengue fever? If so, what would be the discomfort of dengue fever? Could you list symptoms of dengue fever? Do you think dengue fever is terrible or dangerous? Why?
10. Is there the word dengue fever in your ethnic group language? If so, what is dengue fever called in your ethnic group language? What does it mean?
11. In the case that one of the family members had a fever that was suspected possibly to be DF, what the family usually do, and where they usually seek for treatments. Why?
12. Do you know what causes dengue fever? For example, bacteria, viruses, insects, poor air or poor water, etc. If it's caused by these things, will people in contact with them get dengue fever?
13. Is dengue fever contagious? If so, through what? Will dengue patients be directly infected in contact with others? (discuss the relationship between mosquitoes and dengue fever. If no mosquito is mentioned, skip to question.
14. If the answer is Mosquito transmission, when will the mosquito that bites people spread dengue fever? For example, do they bite in the daytime or at night? Or is it stinging day and night?
15. In your opinion, where are there more mosquitoes? Why? Where do mosquitoes usually grow? Do you know that mosquitoes have larvae? If so, where do they grow?
16. Do you know how to prevent mosquito bites and eliminate mosquitoes? Do you know that you can reduce mosquitoes by cleaning up dumps and turning containers upside down and pouring containers and cleaning up the water in them?
17. Do you have any people who often organize to clean the indoor and outdoor environment, such as cleaning up dumps and turning containers upside down, and removing the small water in the indoor and outdoor containers? If so, who organizes and participates?
18. Do you use a water tank or water tank to fill water at your home? If it is used, how often do you change the water and scrub it?
19. Do most people pickle pickles here? If pickled, is the jar mouth covered with plastic bags? If not, how often do you change the water of jar?
20. Do you think the local people know about dengue prevention? What are their attitudes (beliefs) and behaviors towards the prevention of dengue fever? Is it active participation? Why (in-depth study of cultural, socio-economic development, etc.)?
