# Supplementary material for: Treatment seeking behavior and associated factors of suspected dengue fever among Shan people in eastern Shan special region IV, Myanmar: a cross-sectional study
Source: BMC Health Serv Res. 2020 Apr 16;20:318. doi: 10.1186/s12913-020-05163-z (PMC7164341; doi:10.1186/s12913-020-05163-z)
Supplement: Supplementary file 2 — Additional file 2. [file 12913_2020_5163_MOESM2_ESM.docx]

**The questionnaire of household survey ID：**

| **No.** | **Questions** | **Answer candidate** | **No. answer selected** |
| --- | --- | --- | --- |
| 1 | Date（Y-M-D）： |  |  |
| 2 | site: |  |  |
| 3 | Ethnic group | 1）Dai/Shan,2）Wa,3）Jingpo/ Kachin |  |
| 4 | Nationality | 1）China；2）Myanmar |  |
| 5 | Gender | 1 M；2 F |  |
| 6 | School education years | 1）illiteracy；2）1-3 years；3）4-6years；4）7-9 years；5) >= 10 years |  |
| 7 | **Family wealth index** | 1）；2）；3）；4）；5） |  |
|  | \| Family wealth index \| Housing characteristics \| Transportation tools \| Family belongings \| \| --- \| --- \| --- \| --- \| \| 1.Most poor \| Bamboo walls and sheet iron roofs \| None \| None or chickens \| \| 2.Lower mid \| Wood walls and sheet iron roofs \| Bicycles \| Pigs or goats \| \| 3.mid \| Brick walls, wood girders and terracotta roofs \| Motorcycles \| Cattle or horses \| \| 4.higher mid \| Brick concrete walls and terracotta roofs \| Tractors \| TV sets or refrigerators \| \| 5.least poor \| Steel and concrete \| Cars \| Shops or elephants \| | |  |
| 8 | Do you think people with poor economic conditions and poor nutrition are more likely to get sick？ | 1）Y；2）N |  |
| 9 | Do you think you will get sick because of retribution if you don't honor the old, do bad things, etc？ | 1）Y；2）N |  |
| 10 | What religion do you believe in？ | 1）Buddhism，2）Catholicism，  3）Christianity; 4）other: |  |
| 11 | Do you think doing good deeds and accumulating virtues will get the blessing of gods and not easily get sick? | 1）Y；2）N。 |  |
| 12 | Do you think natural environmental factors such as climate, weather, water and forest will affect people's health？ | 1）Y；2）N |  |
| 13 | If so, what kind of natural environment is more likely to get sick? （multiple choice）？ | 1）too hot；2）too cold；3）Too much rain；4）surrounding trees；5）river or pond；6）sewage ditch or odor pond；7）other: |  |
| 14 | Do you think it's easier to get sick because of poor sanitation? | 1）Y；2）N |  |
| 15 | What sanitary conditions are less likely to get sick? (multiple choice）？ | 1）clean；2）no sewage；3）trees and flowers，4）other |  |
| 16 | If it is to do a good job in environmental sanitation, is it not easy to get sick? | 1）Y；2）N |  |
| 17 | Have you heard of dengue fever? | 1）Y；2）N |  |
| 18 | If you've heard about dengue fever, what's the symptoms（multiple choice）? | 1) Fever,2) Headache, 3) orbital pain, 4) systemic pain, 5) rash, 6) others |  |
| 19 | Is dengue fever a severe disease? | 1）Y；2）N |  |
| 20 | Is dengue fever terrible? | 1）Y；2）N |  |
| 21 | will dengue fever be dead? | 1）Y；2）N |  |
| 22 | What causes dengue fever? | 1) bacteria; 2) virus; 3) insect;  4) Others:; 5) do not know or not answer |  |
| 23 | If it is caused by the above things, will the people who bring these things have dengue fever? | 1）Y；2）N |  |
| 24 | If you have fever and headache, what do you usually do first? | 1) using self medication;2) seeking treatment in public hospitals;3) using traditional medication;4) using others;5) did not take any action |  |
| 25 | Do you think it's easy for people to get dengue now? | 1) It's easy,2) Easy, 3) not easy, 4) impossible, 5) other, 6) don't know. |  |
| 26 | Do you think dengue is a infected disease? | 1）Y；2）N |  |
| 27 | If it can, through what? | 1) Flies, 2) mosquitoes, 3) pigs and dogs;4) Drink cold water; 5) get wet or take a cold bath; 6) eat unclean or inappropriate food;7) Others:; 8) do not know or no answer |  |
| 28 | Will people pass dengue fever directly to others? | 1）Y；2）N |  |
| 29 | If mosquitoes transmit dengue fever, when do you think the mosquitoes of dengue fever bite people? | 1) Day; 2) night; 3) all day; 4) don't know or answer. |  |
| 30 | Is the Aedes that transmits dengue fever? | 1）Y；2）N |  |
| 31 | Did you know that mosquitoes grew in water when they were larvae? | 1）Y；2）N |  |
| 32 | Do mosquito larvae that transmit dengue fever mainly grow in bamboo tubes, tree holes, water storage tanks, pickled vegetable jars, broken bottles, cans, rotten pots and old tires with accumulated water? | 1）Y；2）N |  |
| 33 | Are there bamboo tubes and / or tree holes near your home? | 1）Y；2）N |  |
| 34 | If so, have you ever thought about cutting out bamboo tubes and tree holes or filling them with soil to prevent them from accumulating water? | 1）Y；2）N (this is attitude) |  |
| 35 | Do you have any old tires, broken bottles, cans or cans that are easy to accumulate water near your house? | 1）Y；2）N (this is attitude) |  |
| 36 | If so, have you ever thought about getting rid of them? | 1）Y；2）N (this is attitude) |  |
| 37 | How many days do you usually clean your house (fill in specific days)? |  |  |
| 38 | How many days do you usually clean the surrounding environment (fill in specific days)? |  |  |
| 39 | When cleaning the environment, do you turn containers upside down to clean up the water in the container? | 1）Y；2）N |  |
| 40 | Do you have any water tanks or tanks for water? | 1）Y；2）N |  |
| 41 | If so, do tanks and jars have lids or nets? | 1）Y；2）N |  |
| 42 | If so, how many days to change water for cleaning (fill in specific days)? |  |  |
| 43 | Do you have jars for pickles? | 1）Y；2）N |  |
| 44 | If so, is the jar mouth covered with plastic bags or net covers? | 1）Y；2）N |  |
| 45 | If yes, how many days to clean and replace the water in the jar (fill in specific days)? |  |  |
| 46 | Do you have any vase / pot plants such as Phyllostachys pubescens (evergreen) or soil plants with pot supports? | 1）Y；2）N |  |
| 47 | If yes, how many days to clean and change water (fill in specific days)? |  |  |
| 48 | Do you know how to prevent mosquito bites? (multiple choice) | 1) Use Salmonella or screen;  2) Use mosquito repellent incense; 3) apply insecticide; 4) use mosquito net; 5) others; 6) don't know |  |
| 49 | Do you have screen doors and windows? | 1）Y；2）N |  |
| 50 | Do you use mosquito nets? | 1）Y；2）N |  |
| 51 | When you go to a tree (bamboo) forest or a park, will you be exposed with applying mosquito repellent? | 1) often, 2) sometimes, 3) not, |  |
| 52 | Do you know how to reduce or eliminate mosquitoes (multiple choice)? | 1) Using mosquito repellent; 3) using insecticide; 4) to do a good job in environmental sanitation; 5) removing accumulated water; 6) to clean the water tank regularly and replace the water in the tank; 7) don't know |  |
| 53 | Do you know that removing stagnant water, cleaning water tanks regularly and changing water tanks can reduce mosquitoes? | 1）Y；2）N |  |
| 54 | What is the main income of your family? | 1) Planting; 2) working; 3) getting wages; 4) doing business; 5) other:; 6) refusing to answer. |  |
| 55 | Who makes decisions about important matters in your family? | 1) Do not know or answer;  2) Husband; 3) wife; 4) old man; 5) joint consultation; 6) others |  |
